# Unusual case of cavitary lung metastasis from squamous cell carcinoma of the uterine cervix

**DOI:** 10.11604/pamj.2013.14.37.1420

**Published:** 2013-01-27

**Authors:** Soundouss Raissouni, Rais Ghizlane, Houda Mouzount, Kharmoum Saoussane, Setti Khadija, Fouad Zouaidia, Rachida Latib, Hind Mrabti, Hassan Errihani

**Affiliations:** 1 Department of medical oncology, National Institute of Oncology, Rabat, Morocco; 2 Department of pathology, Centre Hospitalier IBN SINA, Rabat, Morocco; 3 Department of Radiology, National Institute of Oncology, Rabat, Morocco

**Keywords:** cavitary lung metastasis, squamous cell carcinoma, uterine cervix

## Abstract

Spontaneous excavation of primary lung cancer is common; however cavitation of metastatic lung lesions is rare and usually confused with benign lesions. In Moroccan context tuberculosis is the first suspected diagnosis of lung excavations. We report a rare case of cavitary lung metastasis of a uterine cervix cancer, treated initially as tuberculosis. A 40-year old non-smoking woman with a known history of squamous cell carcinoma of the uterine cervix since August 2005; presented on September 2008 with right chest pain without fever, hemoptysis or weight loss. CT scan showed a thin walled cavity. Empirical Antibiotic therapy was conducted 15 days with poor outcome. Then antibacillary treatment was started with no proof of mycobacterial infection. A month later, the patient presented with gynecological bleeding and a pneumothorax. Bronchoscopy with transbronchial biopsy of the cavitary mass was performed. Pathology demonstrated a metastatic squamous cell carcinoma. Pelvic examination and MRI showed a subsequent local cervix recurrence. Patient underwent 3 courses of systemic chemotherapy. She died on June 2009 due to progressive disease. Even cavitary lung metastases are rare and benign differential diagnosis are more common, clinician should be careful in neoplastic context and investigation should be done to eliminate a recurrence.

## Introduction

Lung metastases are common and represent 20 to 52% of all metastases sites [1]. Spontaneous cavitation of primary lung cancer is frequent however cavitations of secondary ones are rare. It represents only 4% and usually confused with cavitary benign lesions as infectious, immunologic or systemic disorders [2]. Tuberculosis is endemic in Morocco with an incidence of 82 new cases per 100000 habitants [3]. It is the major cause of cavitary lung lesions in our context. We report here a case of unusual form of lung metastasis of squamous cell carcinoma of uterine cervix treated initially as tuberculosis.

## Patient and observation

**Figure 1:**
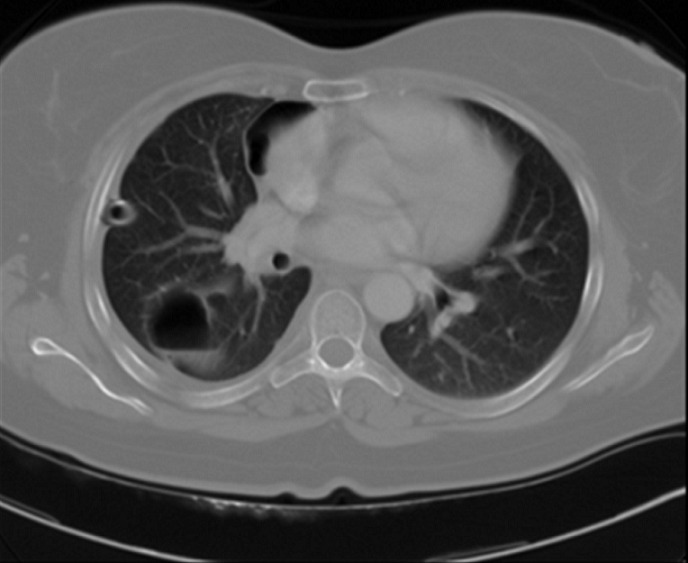
Excavated lesions with clear limits and irregular thin walled

**Figure 2:**
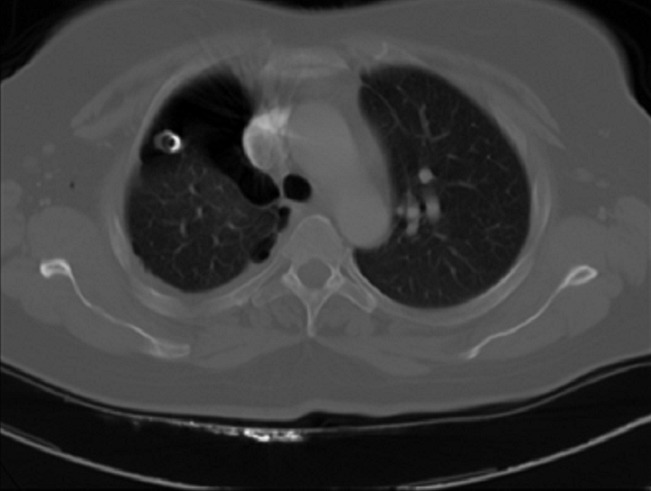
Pneumothorax complicating cavitary lesion

**Figure 3:**
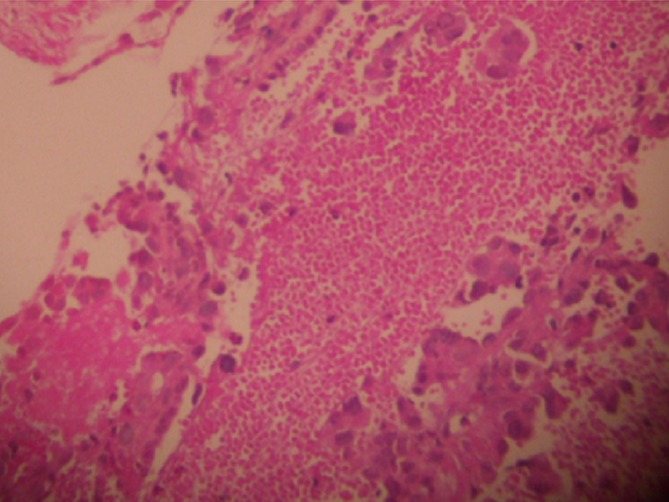
Squamous cell carcinoma with proliferation largely necrotic, made of massive and guts of polygonal cells with hyperchromatic nuclei and abundant eosinophilic cytoplasm (H & Ex 200)

A 40-year old non-smoking woman was followed since august 2005 at the National Institute of Oncology in Rabat, for squamous cell carcinoma of the uterine cervix. It was classified stage IIB according to FIGO classification. Initial treatment consisted of concurrent chemoradiation with 46 grays to the pelvis and weekly platinium based chemotherapy. She was followed every three months with clinical examination, abdominal ultrasound and chest radiograph without any signs of reccurence. She presented on September 2008 with right chest pain without fever, hemoptysis or weight loss. Clinical investigation showed a cavitary lung lesion on CT with a thin walled cavity of 29 mm and wall < 1, 5 mm (Figure 1). Antibiotic therapy with amoxicillin and clavulanic acid was conducted 15 days with poor outcome. Then antibacillary treatment was started with no proof of mycobacterial infection (tuberculin skin test 12 mm, negative sputum culture). A month later the patient presented with leucorrhea and metrorrhagia associated with pneumothorax that required a placement of intercostals chest drain (Figure 2). Bronchoscopy with transbronchial biopsy of the cavitary mass in the right lower lobe was performed. Pathology demonstrated a metastatic squamous cell carcinoma (Figure 3). Clinical examination found a pelvic mass that was confirmed by MRI showing a subsequent local recurrence. On February 2009, the patient underwent 3 courses of systemic chemotherapy with ciplatin 50 mg/m^2^ every three weeks, with acceptable side effects; no improvement in thoracic symptoms was noticed under treatment. She died on June 2009 due to progressive disease.

## Discussion

The presence of cavitation in primary lung cancer is common. It has been reported in 22% [4] and could be a prognostic factor [5]. However pulmonary metastases can rarely cavitate, with a reported incidence less than 4% on chest radiographs [2].

In daily practice, most cavitary lung lesions are benign. The most frequent etiologies include abscess, mycobacterial and fungal infections and immunologic disorders [6]. It could represent a differential diagnosis even in neoplasic context.

Cavitary lung metastasis was first described by Bristowe (1871) who reported a cavitation in a pulmonary metastasis from pharynx carcinoma, diagnosed at necropsy [7]. Most isolated metastatic cavitary lung lesions are squamous cell carcinoma that represents two thirds [2], especially from head and neck [8]. Metastatic adenocarcinomas and sarcomas may also cavitate [1]. The primary sites includes in order of frequency colorectal, pancreas, and lung cancer [9]. Metastasis from uterine cervix cancer is less frequent. Three cases have been reported in an ancient series of 25 cases, and then only few case reports were published [7].

CT scan is more accurate than chest radiographs to detect cavitary lesions. With CT, cavitations in metastatic adenocarcinomas are frequently encountered (9.5%) [1]. Squamous cell carcinoma metastases tend to be thick-walled, cavitating lesions, whereas adenocarcinomas and sarcomas may result in thin-walled cavities. Pneumothorax may develop as a complication as it was the case in our patient [2].

In the present observation, since tuberculosis is endemic in our country and cavitary lesions are found in 40 to 50% [5] it was the first possible diagnosis, that's why antibacillary treatment was conducted with no microbiological proof. Moreover our patient was not smoking and there were no symptoms of uterine cervix recurrence at the beginning.

## Conclusion

Cavitary lung metastases are rare and usually confused with non-neoplasic lesions. In endemic countries, even though tuberculosis is the first cause of cavitary lung lesions, clinicians should be aware of such atypical form of metastases.
